# Effect of Secondary-Phase Precipitation on Mechanical Properties and Corrosion Resistance of 00Cr27Ni7Mo5N Hyper-Duplex Stainless Steel during Solution Treatment

**DOI:** 10.3390/ma15217533

**Published:** 2022-10-27

**Authors:** Hang Wang, Aiqin Wang, Changyi Li, Xingsheng Yu, Jingpei Xie, Chenlu Liu

**Affiliations:** 1 School of Material Science and Engineering, Henan University of Science and Technology, Luoyang 471023, China; 2 Luoyang CITIC HIC Casting & Forging Co., Ltd., Luoyang 471039, China; 3 CITIC Heavy Industries Co., Ltd., Luoyang 471039, China

**Keywords:** hyper-duplex stainless steel, secondary-phase precipitation, microstructure, mechanical properties, corrosion resistance

## Abstract

In this work, the effect of secondary-phase precipitation on the microstructure, mechanical properties, and corrosion resistance of 00Cr27Ni7Mo5N hyper-duplex stainless steel (HDSS) during solution treatment was investigated. The results reveal that σ-phase precipitates at the interface between the α and γ phase when the solution treatment temperature is lower than 1070 °C. It is not only brittle, but also prone to create a Cr-depleted zone, which significantly deteriorates the mechanical properties and corrosion resistance. With the increase in the solution treatment temperature, the volume fraction of ferrite gradually increases. The yield strength and tensile strength increase slightly, but the elongation decreases. At the same time, the impact toughness shows a trend of first increasing and then decreasing. When the solution treatment temperature is higher than 1130 °C, Cr_2_N precipitates in the ferrite. The precipitation of Cr_2_N causes a decrease in the plastic toughness, but it does not deteriorate the mechanical properties as significantly as the σ phase. However, it can also cause the formation of a Cr-depleted zone that significantly decreases the corrosion resistance. There is no secondary-phase precipitation in the sample after solution treatment at 1100 °C, which shows the best mechanical properties and corrosion resistance.

## 1. Introduction

Due to its high strength, stress corrosion resistance, and excellent weldability, super-duplex stainless steel is widely used in the marine industry, petrochemical industry, paper-making industry, and others [1,2,3,4]. In order to cope with severe corrosive working conditions, such as flue gas desulfurization and deepwater oilfield development, 00Cr27Ni7Mo5N HDSS with higher alloy elements came into being. With pitting resistance equivalent values up to 48 or more, it can be used in seawater, high chlorine, and high acid environments for long periods of time [1,4,5]. At the same time, HDSS has excellent mechanical strength, so it can replace the expensive nickel-based alloys. However, the incorporation of a higher content of Cr, Ni, Mo, and nitrogen elements brings excellent strength and corrosion resistance, but increases the risk of precipitation of secondary phases, such as the σ phase in the ternary Fe-Cr-Mo system, χ phase in the quaternary Fe-Cr-Ni-Mo system, and non-equilibrium nitrides [5,6,7,8,9,10,11,12,13,14]. Once precipitated, it can significantly deteriorate the mechanical properties of the alloy as well as the corrosion resistance under harsh conditions [15,16,17].

The increase in the alloying degree of HDSS raises the risk of segregation of Cr and Mo elements, leading to the precipitation of secondary phases more easily, which can significantly deteriorate the mechanical properties and corrosion resistance [1,4,6]. In addition, a higher content of nitrogen can increase the risk of non-equilibrium nitride precipitation [1,18,19], so it is necessary to study the precipitation behavior. The solution treatment temperatures not only affect the diffusion and distribution of alloy elements, grain size, and the transformation between the ferrite (α) and austenite (γ) phase, but also affect the precipitation behavior of secondary phases. An appropriate solution treatment process can not only eliminate harmful secondary phases, but can also rationally distribute Cr, Mo, Ni, Mn, and other elements to achieve an ideal two-phase ratio, which is particularly important for achieving excellent strength and corrosion resistance. For the HDSS with a higher alloy content, the research mainly focuses on the optimization of composition design and the welding process. However, there are few studies on the precipitation behavior of intermetallic phases, non-equilibrium nitrides, and the effect on the comprehensive properties during solution treatment, so more basic studies are urgently needed. Recently, research on the influence of heat treatment on the mechanical properties and corrosion resistance of 20Cr-2Ni-3Mn-0.17N-0.31Mo duplex stainless steel was carried out [20]. The results showed that the tensile strength, yield strength, and pitting resistance of the alloy decreased with the solution treatment temperature increasing from 1000 to 1150 °C. With the increase in the annealing temperature, the pitting resistance equivalent number (PREN) of the α phase decreased, while that of the γ phase increased. The pitting mainly occurred in the α phase. Zhang et al. [21] studied the effect of heat treatment temperature on the microstructure and corrosion behavior of 2101 lean duplex stainless steel. The results indicated that the volume fraction of the γ phase decreased with the solution treatment temperature increasing from 1000 to 1200 °C. With the increase in the heat treatment temperature, the PRE value of the α phase decreased, while the PRE value of the γ phase increased. Pitting corrosion mainly occurs at the α/γ phase boundary or within the α phase. Moreover, Gao et al. [22] studied the effect of annealing temperature on the microstructure and corrosion behavior of 2507 duplex stainless steel. The results showed that the volume fraction of the γ phase decreases with the increase in the annealing temperature. The scanning Kelvin probe force microscopy (SKPFM) measurements showed that the voltage potential of the γ phase was higher than that of the α phase. The in situ atomic force microscopy (AFM) observation proved that the corrosion occurred preferentially in the α phase. The sample annealed at 1150 °C exhibited a smaller voltage potential difference and a lower corrosion rate. It can be seen that the microstructure and composition of DSS can affect its mechanical properties and corrosion resistance greatly.

In order to expand the application of HDSS, it is very important to obtain excellent corrosion resistance and mechanical properties. In this work, the effect of secondary-phase precipitation on the mechanical properties and corrosion resistance of 00Cr27Ni7Mo5N HDSS during solution treatment was investigated. The results showed that there was no secondary-phase precipitation in the sample after the solution treatment at 1100 °C, which exhibited the best corrosion resistance and mechanical properties. While the precipitation of the secondary phases during solution treatment was not significant in previous studies, our study found that the precipitation of metallic phases and non-equilibrium nitrides had a significant effect on the mechanical properties and corrosion resistance of the HDSS with a higher alloy content. The work is of great help to industrial applications as it provides theoretical guidance for the formulation of heat-treatment processes.

## 2. Materials and Methods

**Figure 1:**
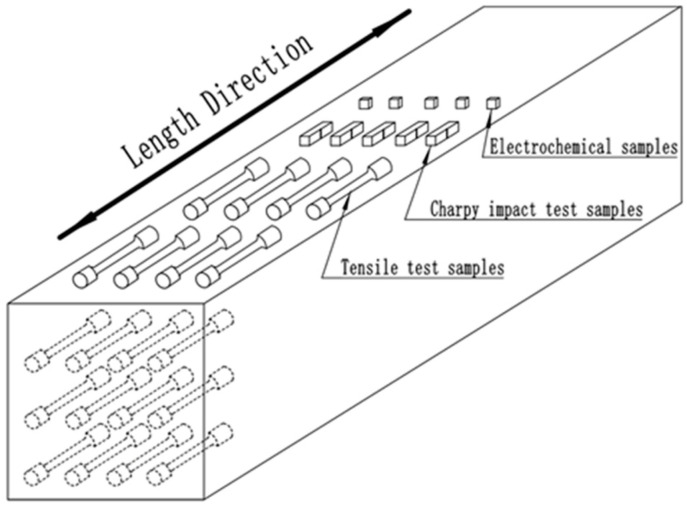
Sampling schematic for test specimens.

The experimental alloy was melted and poured into 50 kg ingots in a vacuum induction furnace (ZG-100 type, Suzhou Zhenwu Electric Furnace Co., Ltd., Suzhou, China) with the chemical composition of 0.023 ± 0.002 C, 0.46 ± 0.02 Si, 1.19 ± 0.04 Mn, 27.01 ± 0.11 Cr, 6.51 ± 0.08 Ni, 4.54± 0.04 Mo, 0.92 ± 0.04 Cu, 0.99 ± 0.03 Co, 0.42 ± 0.01 N (in wt.%), and Fe balance. The experimental steel ingot was forged into billets of 80 mm in length and 80 mm in width. The cross section of samples was processed to 14 mm × 14 mm. The sampling schematic for the test specimens is shown in Figure 1. In order to study the effect of different solution temperatures on the microstructure evolution, mechanical properties, and corrosion resistance, the specimens were solution-treated at 1040, 1070, 1100, 1130, 1150, 1180, and 1200 °C for 80 min, respectively, and then quenched in water rapidly.

The specimens were etched with 2 g of potassium metabisulfite, 30 mL of hydrochloric acid, and 100 mL of water solution for 2~5 min. Ten fields of view were selected for each sample, and MIAPS software was used to measure the phase fraction in the tissue. The scanning electron microscope (ZEISS EVO-18, Carl Zeiss AG, Jena, Germany) was used for fracture morphology, energy spectrum analysis, and pitting corrosion morphology analysis. The nitrogen content was analyzed using an electron probe micro-analyzer (JXA-8530F Plus, Japan Electronics Co. Ltd., Tokyo, Japan). The samples for transmission electron microscopy (TEM) analysis were electrolytically double-spray polished in 5% perchloric acid ethanol solution at -20 °C and 50 V. The TEM analyses were performed using transmission electron microscope (JEM-2100, Japan Electronics Co. Ltd., Tokyo, Japan) at 200 kV. The mechanical properties of the samples were examined according to the standard ASTM A370 at room temperature. Cylindrical specimens measuring 6.25 mm in diameter and 25 mm in gauge lengths were used to test the tensile properties by 2 mm/min on the testing machines (DDL300, Sinotest Equipment Co., Ltd., Changchun, China). The V-notched specimens (55 mm × 10 mm × 10 mm) were used to measure the Charpy impact on the testing machine (NI150C, NSC Testing Technology Co., Ltd., Beijing, China).

The potentiodynamic anodic polarization tests were determined using an electrochemical workstation (Autolab PGSTAT128N, Metrohm AG, Herisau, Switzerland). The tests were performed in a de-aerated 3.5% NaCl solution at room temperature. A three-electrode system was used, with a test specimen of 1 cm^2^ exposed area as the working electrode, a large area graphite electrode as the auxiliary electrode, and a saturated calomel electrode as the reference electrode. The scanning rate was set at 20 mV/min. The scanning range was −600 mV_SCE_ to 1200 mV_SCE_.

The mechanical properties tests and potentiodynamic anodic polarization tests were determined by means of the average values of three specimens. The data were reported as mean ± standard deviation values.

## 3. Results and Discussion

### 3.1. Effect of Solution Treatment Temperatures on Microstructure

**Figure 2:**
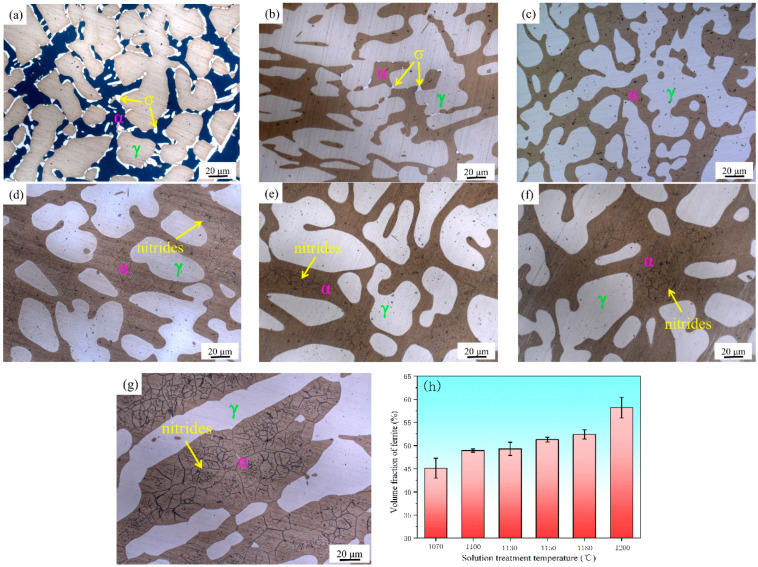
Microstructural evolution of samples with different solution treatment temperatures at: (**a**) 1040 °C; (**b**) 1070 °C; (**c**) 1100 °C; (**d**) 1130 °C; (**e**) 1150 °C; (**f**) 1180 °C; and (**g**) 1200 °C; (**h**) volume fraction of α phase.

Figure 2 shows the microstructural evolution of the samples with different solution treatment temperatures, in which the island-shaped light white γ phase is distributed in the gray α-phase matrix. Figure 2a shows that a large amount of undissolved σ phase exists at the boundary between the α and γ phase after solution treatment at 1040 °C. There are still small amounts of σ phase at 1070 °C (Figure 2b). When the solution treatment temperature is higher than 1100 °C, the σ phase can be completely eliminated (Figure 2c). The grain size of the ferrite begins to coarsen when the solution treatment temperature is higher than 1130 °C. The proportion of nitrides in the α phase gradually increase (Figure 2d–g). However, there is no secondary-phase precipitation in the γ phase. With the increase in the solution treatment temperature, the volume fraction of the α phase gradually increases, as shown in Figure 2h. A typical diffusion-controlled solid-phase transformation occurs, with a ratio of nearly 50% in the temperature range of 1100 to 1150 °C.

**Figure 3:**
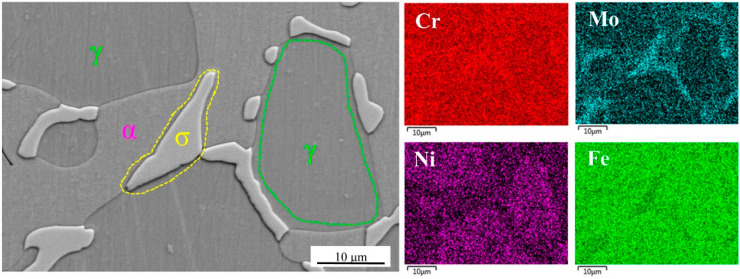
SEM image and EDS elemental distribution assessment of the function of Cr, Mo, Ni, and Fe of each specimen after solution treatment at 1040 °C.

**Figure 4:**
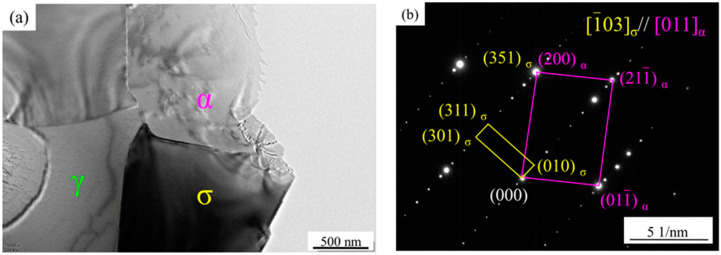
TEM images (**a**) and SAED patterns (**b**) of precipitates of the specimens after solution treatment at 1040 °C.

The SEM image and EDS analysis of the sample after solution treatment at 1040 °C are shown in Figure 3. It can be seen that the secondary phase is rich in Cr and Mo elements. The TEM images and electron diffraction (SAED) analysis of the secondary phase with lattice constants a = b = 0.879 nm and c = 0.454 nm (Figure 4), which is identified as σ phase. The orientation relationship between the α and σ phase can be determined as follows:

(1) $${\left[\bar{1}03\right]}_{\sigma }∥{\left[011\right]}_{\alpha }\;\mathrm{and}\;{\left(351\right)}_{\sigma }∥{\left(200\right)}_{\alpha }$$

It has been shown that the mechanism of σ-phase precipitation is a co-precipitation transition of α phase: α→σ + γ_2_ [15,23,24,25]. The diffusion rate of alloy elements in the α phase is relatively high. The σ phase initially nucleates at the phase boundaries, and then grows toward the inner ferrite, enriching the Cr and Mo elements. The growth of σ-phase precipitation further consumes the Cr and Mo elements in the ferrite, so the ferrite phase with high Ni content becomes unstable and eventually transforms into the γ_2_ phase. HDSS has a higher content of Cr and Mo elements, which increases the risk of segregation and shortens the σ phase formation gestation period. Therefore, it is more favorable for σ-phase nucleation and growth.

**Figure 5:**
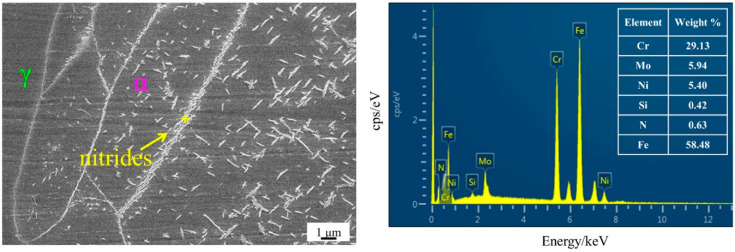
SEM-EDS analysis of specimen after solution treatment at 1200 °C.

**Figure 6:**
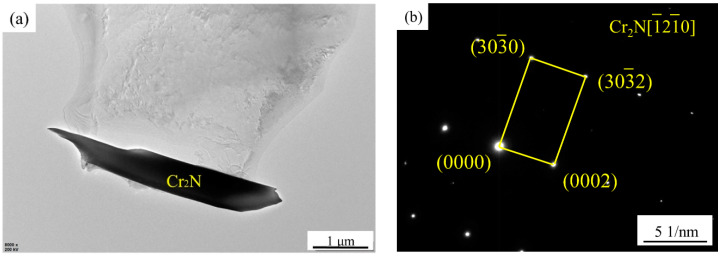
TEM images (**a**) and SAED patterns (**b**) of precipitates of the specimens after solution treatment at 1200 °C.

Figure 5 shows the SEM image and EDS analysis of the sample after solution treatment at 1200 °C. A large number of long rod-like secondary phases precipitate, which are concentrated inside the α phase and the grain boundary between the α phase. The EDS analysis shows that it is rich in Cr and nitrogen elements, among which the content of Cr elements is higher than that of the matrix. Figure 6 show the TEM analysis of Cr_2_N, which is determined as a hexagonal close-packed (hcp) structure. The lattice parameters are a = 0.480 nm and c = 0.447 nm. Liang et al. [14] found the presence of nanorod chromium nitride precipitation on lamellar carbides along the α- and γ-phase boundary in UNS S32760 super-duplex stainless steel. Shi et al. [18] reported that Cr_2_N showed granular and lamellar morphology, while Zhang et al. [19] reported that the shape of Cr_2_N was speckled. This work shows that the Cr_2_N precipitated in HDSS exhibits long rod-like structures. With the increase in the solution treatment temperature, the solid solubility of nitrogen elements in the α phase increases rapidly, and a large number of nitrogen atoms migrate to the α phase, which cannot be diffused in time during the water-cooling process, so they are dispersed and precipitated in situ in the α phase [19,26]. Deng et al. [27] indicated that non-equilibrium nitrides can only precipitate within the α phase in UNS S32750 super-duplex stainless steel when the solution treatment temperature is higher than 1200 °C. In this work, the content of the nitrogen element in HDSS is as high as 0.43 wt.%. These kinds of nitrides are precipitated in the α phase after solution treatment at 1130 °C, which reveals that the increase in the nitrogen content significantly promotes the precipitation of non-equilibrium nitrides.

### 3.2. Effect of Secondary-Phase Precipitation on Mechanical Properties

**Figure 7:**
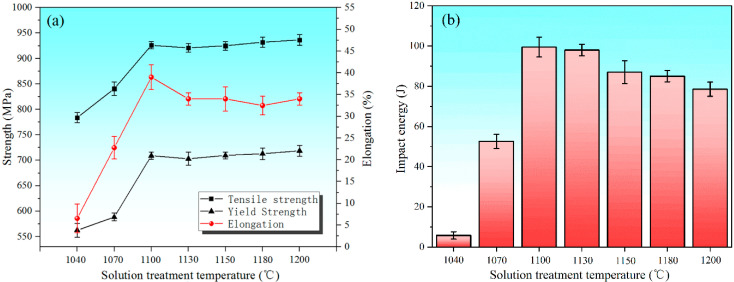
Mechanical properties of specimens with different solution treatment temperatures: (**a**) tensile property and (**b**) Charpy impact energy.

Figure 7 shows the tensile strength and impact test results of samples with different solution treatment temperatures. It can be seen from Figure 7a that the tensile strength and plasticity of the specimens is extremely low after solution treatment at 1040 °C, where the elongation is 7 ± 3% and the impact toughness is 6 ± 2 J. With the increase in the solution treatment temperature up to 1100 °C, the mechanical properties are substantially improved, in which the elongation and impact toughness is 39 ± 3% and 100 ± 5 J, respectively. When the solution treatment temperature is higher than 1100 °C, the yield strength and tensile strength increase slightly, but the elongation decreases. Meanwhile, Figure 7b shows that the impact toughness of the specimens increases first and then decreases with the increase in the solution treatment temperature.

**Figure 8:**
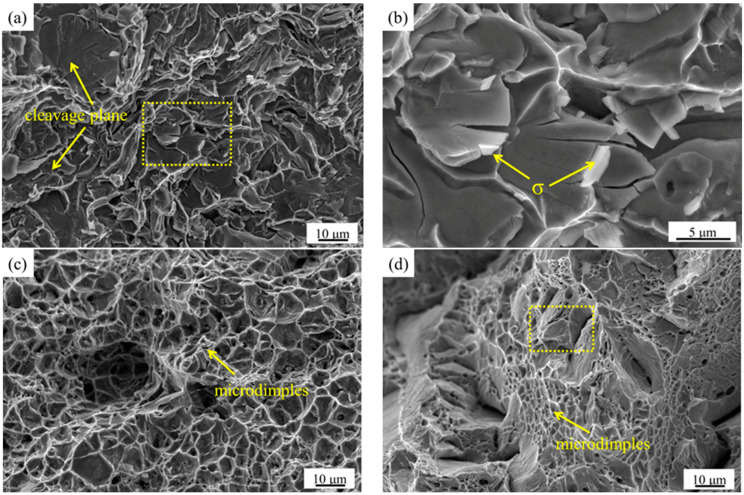

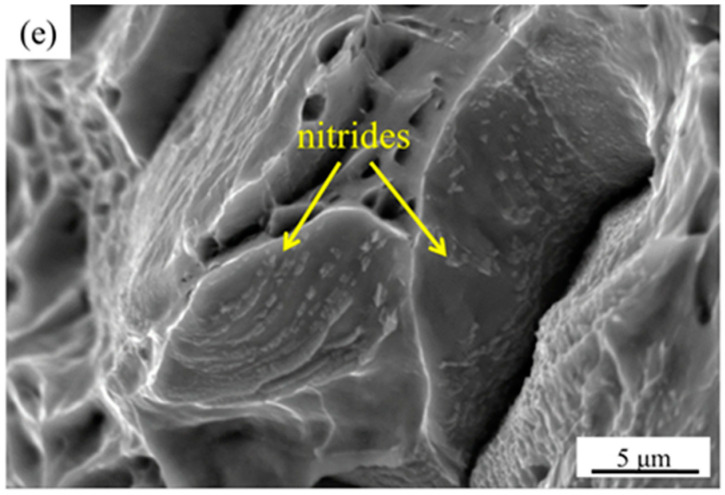
The SEM images of tensile fracture of the specimens after solution treatment at: (**a**) 1040 °C; (**b**) enlarged images of square frame area in (**a**); (**c**) 1100 °C; (**d**) 1200 °C; (**e**) enlarged images of square frame area in (**d**).

The SEM images of tensile fracture at typical solution treatment temperatures are shown in Figure 8. It can be seen from Figure 8a,b that the tensile fracture of the sample is mainly a mixture of cleavage fracture and fine dimples after the solution treatment at 1040 °C, which is basically brittle fractures. A large amount of massive σ phase exists on the fracture and tear marks (Figure 8b). Figure 8c shows that the tensile fracture of the sample presents a ductile fracture with a large number of deep dimples after solution treatment at 1100 °C. The tensile fracture equiaxed dimples gradually decrease, and a large number of micron-level nitrides attach to the fracture at 1200 °C (Figure 8d,e).

**Figure 9:**
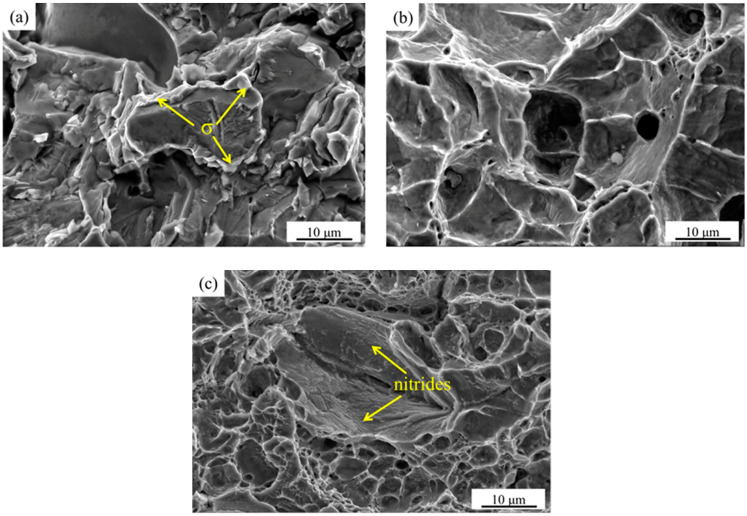
SEM images of impact fracture of the specimens after solution treatment at: (**a**) 1040 °C; (**b**) 1100 °C; (**c**) 1200 °C.

Figure 9 shows the SEM images of impact fracture at typical solution treatment temperatures. Figure 9a shows that the impact fracture of the sample is a typical brittle fracture with a large amount of σ phase surrounding the matrix, resulting in cracking separation between the matrixes at 1040 °C. Figure 9b shows that the impact fracture of the sample is a ductile fracture with a large number of equiaxed dimples after solution treatment at 1100 °C. A large number of micron-level nitrides are attached to the impact fracture of the sample after solution treatment at 1200 °C (Figure 9c).

The solution treatment process not only affects the diffusion and distribution of alloy elements, grain size, and the transformation between the α and γ phase, but also affects the precipitation of the secondary phases. The changes of these factors have a significant impact on the microstructure and mechanical properties of HDSS. The σ phase precipitates at the boundary between the α and γ phase when the sample is solution treated below 1070 °C. Therefore, a large number of dislocations gather around the σ phase, which has hard and brittle characteristics, so cracks tend to initiate around it and then expand rapidly, resulting in brittle fractures under the action of tensile stress [1]. When the solution treatment temperature increases from 1100 to 1200 °C, the size of the grain coarsens. The volume fraction of the α phase increases. The yield strength and tensile strength increase slightly, while the proportion of γ phase is relatively low, resulting in a decreasing trend of elongation. The results show that the α phase is stronger than the γ phase in HDSS, which affects the overall strength, while the γ phase proportion mainly affects the overall plasticity and toughness. In addition, the σ phase that precipitates at the interface of the two phases has almost no plastic deformation under the action of high-speed impact loads, which tends to cause stress concentration and crack initiation, and then rapidly expands to release energy [28,29]. The sharp corner of the long rod-shaped Cr_2_N combined with the matrix (Figure 6a) also tends to form stress concentration under high-speed impact loads, resulting in the decrease in the plasticity and toughness. However, the nitride precipitation size is at the micron level, which has a deteriorating effect on the mechanical properties, but it is far less significant than that of the σ phase. Therefore, HDSS has excellent mechanical properties when solution treated in the range of 1100 to 1130 °C.

### 3.3. Effect of Secondary-Phase Precipitation on the Resistance to Corrosion Resistance

**Figure 10:**
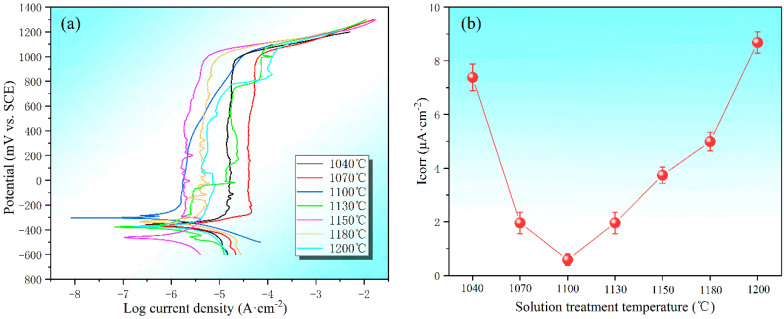
Potentiodynamic anodic polarization curves (**a**) and corrosion current densities (**b**) of the specimens after solution treatment at different temperatures.

Figure 10 shows the potentiodynamic anodic polarization curves and corrosion current densities (Icorr) of the specimens with different solution treatment temperatures. With the increase in the solution treatment temperature, the corrosion current densities show a trend of first decreasing and then increasing (Figure 10b). The Icorr value of the sample is at its lowest (Icorr: 0.59 μA∙cm^−2^) after solution treatment at 1100 °C. However, the highest Icorr value of the sample (Icorr: 8.68 μA∙cm^−2^) is observed after solution treatment at 1200 °C. The corrosion rate of metals is proportional to the Icorr [30]; therefore, the lower the corrosion current density, the slower the corrosion rate, which can be used to describe the corrosion resistance of the material. The current metal corrosion rate index is used to measure the corrosion rate by the anode current density in the electrochemical corrosion process. Because of the defects, inclusions, and solute inhomogeneity of the passive film in the alloy, it becomes very fragile in these places and can be easily damaged. In turn, it becomes an activated anode and forms a primary cell with the surrounding area, which can further develop into pitting corrosion. The precipitation of the σ phase and Cr_2_N can cause the generation of Cr-depleted zones around them, thus triggering selective corrosion and leading to a significant increase in pitting corrosion susceptibility. This implies that the best pitting resistance can be obtained by solution treatment at 1100 °C.

**Figure 11:**
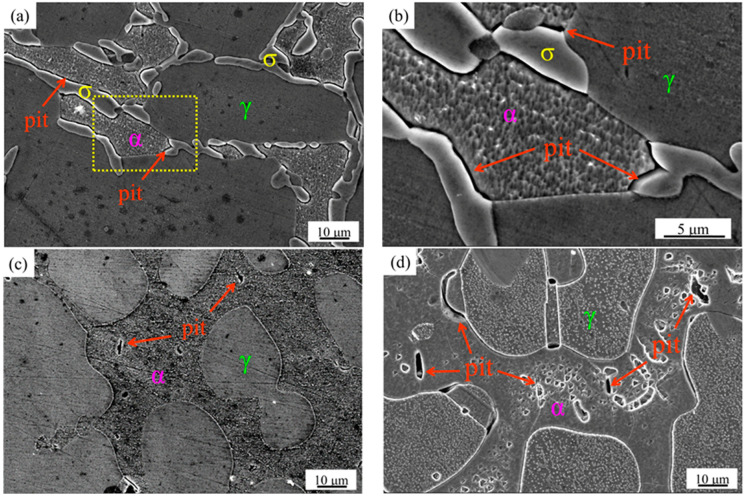
SEM images of the specimens after potentiodynamic anodic polarization test solution treatment at: (**a**) 1040 °C; (**b**) enlarged images of square frame area in (**a**); (**c**) 1100 °C; (**d**) 1200 °C. The pit refers to the area of weak corrosion resistance where the passivation film is damaged by chloride ions and formed by further longitudinal development.

In order to further investigate the corrosion mechanism at different solution treatment temperatures, SEM images of pitting corrosion were obtained (Figure 11). Figure 11a,b show the pitting corrosion morphology of the sample after solution treatment at 1040 °C. It can be observed that there are pits around the σ phase, which precipitates at the interface of the α and γ phase. With the increase in the solution treatment temperature up to 1100 °C, only α and γ phase are presented in the microstructure, which exhibits a few small-sized pits (Figure 11c). Figure 11d shows a significant increase in the number and size of the pits in the α phase of the specimen after solution treatment at 1200 °C. The shape of the pits also basically matches the long rod-like Cr_2_N morphology. In general, the trend change of the Icorr values is consistent with that of the pits.

It is widely known that the typical microstructure of duplex stainless steel is characterized by α and γ phases, where the higher the concentration of Cr, Mo, W, and N elements, the stronger the resistance to corrosion. The evaluation of the corrosion resistance is significantly related to the PREN value and the proportion of the two phases. Therefore, an appropriate solution treatment process should be carried out to reasonably distribute the alloy elements, so as to obtain the best two-phase ratio and achieve the best corrosion resistance. The chemical compositions and PREN values of the α and γ phases of the specimens at different solution treatment temperatures are shown in Table 1. The results show that the Cr and Mo elements are enriched in the α phase, while N elements are enriched in the γ phase. The octahedral interstitial sites of the austenitic face-centered cubic (FCC) lattice are larger than the octahedral interstices and tetragonal interstices of the ferritic body-centered cubic (BCC) lattice. The nitrogen atoms basically occupy the austenitic octahedral interstitial sites, leading to their saturation in the γ phase. Therefore, the nitrogen atom is much more soluble in the γ phase than that in the α phase, which almost completely dissolves in the γ phase. The nitrogen solubility in the α phase has an extremely low content, with a maximum of 0.05 wt.% [31,32]. The PREN values of the γ phase are higher than those of the α phase. As the solution treatment temperature increases, the difference increases. Pitting corrosion tends to occur preferentially in the α phase. In addition, the existence of defects, inclusions, and secondary phases in duplex stainless steels can deteriorate the self-healing ability of the passivation film on the matrix surface and accelerate the corrosion behavior in chloride-corrosive media. Suter et al. [33] reported that the region of highest electrochemical activity was not the metal matrix or inclusions, but the interface between the inclusions and the metal matrix, where pitting corrosion was more likely to occur. Therefore, the secondary phases precipitate during solution treatment, such as σ phase and Cr_2_N, which are the focus of this work, tend to cause the Cr-depleted zone around them, which significantly reduces the corrosion resistance of HDSS.

When the specimen is solution-treated at 1040 °C, a large amount of σ phase precipitates along the α- and γ-phase interface. Although the difference between the PREN values of the α and γ phase is not the largest, related reports [15,16,17] have shown that pitting corrosion tends to occur around the Cr-depleted zone due to the precipitation of σ phase. Therefore, pits tend to develop along the depth of this area, and further expand due to selective corrosion, resulting in worse corrosion resistance. The specimen shows the best corrosion resistance with no precipitation of secondary phase at 1100 °C. After solution treatment at 1200 °C, the sample precipitates a large amount of Cr_2_N, which has a relatively sharp angle shape (Figure 6a). In addition, its expansion coefficient has a large difference with the metal matrix, which makes it easier to form gaps at the interface [34,35]. In the chloride environment, it tends to become the origin of pitting corrosion, and then expands until it is dissolved. The difference in the PREN values between the α and γ phases is the largest at 1200 °C. In addition, the presence of Cr_2_N significantly increases the susceptibility of pitting corrosion, resulting in a significant decrease in corrosion resistance. Therefore, the worst corrosion resistance is exhibited. In this work, the corrosion resistance of HDSS is mainly determined by the presence of secondary phases, such as σ phase and Cr_2_N, volume fractions, elemental distribution behavior, and PREN value differences between the α and γ phases.

## 4. Conclusions

The purpose of this work is to gain insight into the effect of secondary-phase precipitation on the microstructure, mechanical properties, and corrosion resistance of 00Cr27Ni7Mo5N HDSS steel during solution treatment. The results have important implications for guiding industrial applications. The following conclusions have been drawn:

- The best corrosion resistance of 00Cr27Ni7Mo5N HDSS can be obtained when it is solution-treated at 1100 °C. At the same time, it exhibits excellent mechanical properties, which can be better applied to severe corrosive working conditions.
- When the solution treatment temperature of the sample is lower than 1070 °C, σ phase exists in the interface between the α and γ phase, which is brittle and prone to create a Cr-depleted zone around it, significantly deteriorating the mechanical properties.
- There is no secondary-phase precipitation of the sample after solution treatment at 1100 °C. The yield strength and tensile strength increase slightly, but the plastic toughness decreases with the solution treatment temperature increasing from 1100 to 1200 °C, which can be explained by the volume fraction of α phase and the precipitation of Cr_2_N. The precipitation of Cr_2_N does not deteriorate the mechanical properties as significantly as the σ phase.
- With the increase in the solution treatment temperature, the corrosion current densities first decrease and then increase, reaching the lowest value at 1100 °C. The precipitation of σ phase and Cr_2_N can cause the generation of Cr-depleted zones around them, thus triggering selective corrosion and leading to a significant increase in pitting corrosion susceptibility.

## Figures and Tables

**Table 1:** Chemical compositions and PREN values of α and γ phases of the specimens at different solution treatment temperatures.

Solution Temperatures (°C)	Phase	Volume Fraction (%)	Chemical Compositions (Mass %)			PREN ^1^	ΔPREN PREN(γ)-PREN(α)
			Cr	Mo	N		
1040	Ferrite (**α**)	27.2	28.91	6.26	0.05	51.1	7.6
	Austenite (**γ**)	57.8	25.49	3.44	0.73	58.7	
1100	Ferrite (**α**)	49.3	28.48	6.02	0.05	49.9	10.4
	Austenite (**γ**)	50.7	25.52	3.46	0.78	60.3	
1200	Ferrite (**α**)	58.2	27.63	5.76	0.05	48.1	11.4
	Austenite (**γ**)	41.8	25.55	3.57	0.74	59.5	

^1^ PREN = wt.% Cr + 3.3 (wt.% Mo + 0.5 wt.% W) + 30 wt.% N.

## Data Availability

Not applicable.
